# Treatment of pelvic cavity pain caused by endometriosis with excision of invaded sacrospinous ligament

**DOI:** 10.12669/pjms.345.14830

**Published:** 2018

**Authors:** Jinqiong Li, Xia Yao, Jing Zhang

**Affiliations:** 1 Jinqiong Li, Department Gynecology and Obstetrics, Affiliated Heji Hospital of Changzhi Medical College, Changzhi, Shanxi Province, 046000, P. R. China; 2 Xia Yao, Department Gynecology and Obstetrics, Affiliated Heji Hospital of Changzhi Medical College, Changzhi, Shanxi Province, 046000, P. R. China; 3 Jing Zhang, Department Gynecology and Obstetrics, Affiliated Heji Hospital of Changzhi Medical College, Changzhi, Shanxi Province, 046000, P. R. China

**Keywords:** Sacrospinous ligament excision, Endometriosis, Pain

## Abstract

**Objective:**

To evaluate the clinical therapeutic effects of excision of invaded sacrospinous ligament on pelvic cavity pain caused by endometriosis.

**Methods:**

Eighty endometriotic patients treated in our hospital from January 2013 to December 2014 were chosen, and divided into a control group and an observation group. Regular operation (i.e. excision of endometriotic nidus and separation of pelvic cavity adhesion) was performed for the control group, while regular operation and sacrospinous ligament excision were conducted for the observation group. Intraoperative and postoperative conditions as well as postoperative pain remission of both groups were compared.

**Results:**

For the amount of bleeding during operation, the control group was (120±5.2) ml, while the observation group was (160±4.0) ml. For the duration of operation, the control group was (65±3.4) minutes, while the observation group was (92±2.6) min (p<0.05), with a significant difference. For the independent urination time after operation, the control group was (32±8.8) hour, while the observation group was (33±6.4) hour. For the evacuation time after operation, the control group was (38±2.6) hour, while the observation group was (39±3.0) hour (p>0.05), with a significant difference. The postoperative VAS scores of the two groups were significantly lower than those before operation, and the VAS score of the observation group was significantly lower than that of the control group, p<0.05.

**Conclusions:**

Sacrospinous ligament excision relieved pain caused by endometriosis, so it may be applicable to the endometriosis patients with sacrospinous ligament infiltration or severe pain.

## INTRODUCTION

Endometriosis is a common gynecological disease typified by appearance of endometrium in other parts beyond the uterine cavity, leading to pelvic cavity mass, pain and infertility.^1^ Pain is a common symptom of endometriosis, including chronic pelvic cavity pain, dysmenorrhea and dyspareunia. As a result, patients’ living quality as well as physical and psychological healths are severely affected.^2^ Endometriosis can be classified into peritoneal, ovarian and deeply infiltrative types. Deeply infiltrative endometriosis is an important type causing clinical pain. The involved regions cover the ovary, peritoneum, vagina, intestinal canal, ureter and ischiadic nerve. Besides, it also invades uterine-rectal lacuna and sacrospinous ligament, generating other clinical manifestations such as difficult defecation and intestinal irritability.^3^-^4^ Irritating nidus is refractory and prone to relapse. Thus, particular attention has been paid to endometriosis treatment. In this study, we excised invaded sacrospinous ligament to relieve endometriosis-induced pain.

## METHODS

A retrospective study was conducted to select endometriotic patients in our hospital from January 2013 to December 2014, and the patients with sacrospinous ligament infiltration. According to the random draw principle, 40 of the patients were randomly selected from the patients selected by laparoscopy or on the basis of the sacral ligaments. A total of 80 patients were selected as control group and observation group. This study was approved by the ethics committee of our hospital, and written consent has been obtained from all patients. The dysmenorrheal degree was evaluated according to Visual Analogue Scale (VAS) (scoring standard: 0, no pain; 3 and below, mild pain; 4-6, pain which affects sleep but can be borne; 7-10, severe pain difficult to bear). The score was above seven, and that of pelvic cavity pain in the non-menstrual period was above six. The patients with pain induced by pelvic cavity inflammation or other internal and external medical diseases causing stomachache (e.g. intestinal spasm and ileus) were excluded. There was no history of pelvic cavity operation. The 80 patients had indications of operation and received laparoscopic surgery. Intraoperative stages of endometriosis were staged according to the extent of lesion spread, the revised ASRM modified classification system (1997).^5^ Two groups of patients signed preoperative informed consent before surgery. In the control group and the observation group, postoperative sacral ligament delivery for pathological examination returned to varying degrees of the characteristics of endometriosis.

The age of control group was (38.4±7.6) and the duration of disease was (3.5±1.1) years. The age of observation group was (37.8±8.0) and the duration of disease was (3.4±1.0) years. The two groups had comparable ages, pain degree scores and durations of disease.

Laparoscopic surgery was performed for both groups. Regular operation was carried out for the control group, including excision of endometriotic nidus (ovary, oviduct, uterus and peritoneum) and separation of pelvic cavity adhesion. Only endometriotic nidus on the surface of sacrospinous ligament or obvious lesion on the obviously thickening or hardening part was cut off, and the sacrospinous ligament was not completely cut off. For the observation group, sacrospinous ligament excision was conducted on the basis of regular operation. If the sacrospinous ligament had a large lesion, it was partly cut off. Due to severe adhesion of lesion during operation, the hypogastric nerve near sacrospinous ligament may be injured during separation of uterine-rectal lacuna and excision of sacrospinous ligament, so patients may suffer from dysfunction of the rectum and bladder. Thus, the hypogastric nerve should be carefully identified by firstly separating the gap between sacrospinous ligament and rectum, exposing the intersection between ureter and uterine artery, pushing away the ureter, separating downward along the uterine artery and pulling the uterus to the symphysis pubis to expose the hypogastric nerve and branch. The nerve was circumvented to conduct regular operation for deeply infiltrative endometriosis. Briefly, the endometriotic nidus was cut off after separation of pelvic cavity adhesion, and the uterosacral ligament was fully exposed during sacrospinous ligament excision. After the uterus and rectum were cut off and pushed downward, the peritoneum was bent to dissociate the sacrospinous ligament, and the upper end of sacrospinous ligament was cut off by bipolar coagulation or ultrasound knife. If the sacrospinous ligament had endometriotic nidus characteristics such as thickening and hardening, it was cut off till the root. The two groups were compared regarding operation duration, hemorrhage during operation, independent urination time after operation, evacuation time after operation and remission degree of pelvic cavity pain (VAS score).

### Statistical Analysis

SPSS 17.0 software package was used for statistical analysis. Measurement data were expressed as mean ± standard deviation. The *u* test was employed for inter-group comparison. Numerical data were expressed as frequency and percentage. The rank sum test was applied for inter-group comparison. P<0.05 was considered statistically significant.

## RESULTS

Both groups were staged according to the revised ASRM modified classification system as follows. Control group: 0 in Stage I, 1 in Stage II, 26 in Stage III and 13 in Stage IV; observation group: 0 in Stage I, 2 in Stage II, 28 in Stage III and 10 in Stage IV. There was no significant difference between the two groups (p>0.05) (Table-I).

**Table-I:** Different intraoperative stages (n, %).

Group	No.	Stage I	Stage II	Stage III	Stage IV
Control	40	0 (0)	1 (2.50)	26 (65.0)	13 (32.5)
Observation	40	0 (0)	2 (5.00)	28 (70.0)	10 (25.0)
Zc	0.841				
P	0.703				

The two groups had significantly different operation durations and amounts of bleeding during operation (p<0.05). However, their independent urination times after operation and evacuation times after operation were similar (p>0.05) (Table-II). Moreover, comparison of both groups in post-operative pain easement (p<0.05) (Table-III).

**Table-II:** Operation comparison of both groups (*x*±s).

Group	No.	Amount of bleeding during operation (ml)	Operation duration (min)	Urination time after operation (h)	Evacuation time after operation (h)
Control	40	120±5.2	65±3.4	32±8.8	38±2.6
Observation	40	160±4.0	92±2.6	33±6.4	39±3.0
u		38.561	39.896	0.581	1.592
P		0.001	0.001	0.715	0.602

**Table-III:** Comparison of both groups in post-operative pain easement (x±s).

Group	No.	VAS score			

		Before operation	after operation	u	P
Control	40	7.61±0.72	3.22±0.27	4.592	0.017
Observation	40	7.82±0.58	1.15±0.41	9.921	0.001
u	-	0.192	6.629		
P	-	0.681	0.012		

## DISCUSSION

Endometriosis is a common gynecological disease that widely involves the uterus, oviduct, ovary, intestinal canal, etc.^6^,^7^ Pain is the most common clinical manifestation of this disease. Endometriosis can be classified into peritoneal, ovarian and deeply infiltrative types. The nidus of deeply infiltrative type is mainly distributed at uterine-rectal lacuna and sacrospinous ligament. Deeply infiltrative endometriosis is an important type causing clinical pain.^8^ The pain degree caused by endometriosis is related not always to lesion degree, but to lesion infiltration depth, distribution range, adhesion degree and nerve fiber distribution change in the nidus. If the lesion exists in the ovary and endometrial implantation cyst forms, the correlation with clinical pain is low. If the lesion exists in uterine-rectal lacuna or sacrospinous ligament, severe and intolerable clinical pain takes place.^9^-^10^ Endometriosis-induced pain may be ascribed to aseptic inflammation induced by mild periodical bleeding of nidus. It may also be related to pulling of adhesive belt or muscle fiber around the uterus which contracts due to nidus stimulation. In addition, invasion of the pelvic nerve by nidus may also cause pain.^11^ The nerves dominating pelvic cavity, including urinary bladder, rectum, uterus and upper end of vagina, are mainly hypogastric nerve and hypogastric nerve plexus formed by nervus visceralis from the sciatic plexus (s2-s4). Hypogastric nerve is the branch of superior hypogastric nerve, which spans the middle part of sacrospinous ligament and participates in forming hypogastric nerve plexus to dominate urinary bladder, rectum, uterus and so on.^12^ Therefore, during separation and cutting of sacrospinous ligament, the surrounding hypogastric nerve plexus may be easily injured, thereby affecting the recovery of urinary bladder and rectal functions after operation. Nevertheless, endometriosis-induced pain may be relieved due to the injury of hypo gastric nerve plexus.

The main components of sacrospinous ligament include fibrous connective tissue, adipose tissue, blood vessel and rich nerve fibers. Besides, lymph and a few lymph nodes are also included.^13^ Sensory nerves of the uterus and oviduct enter pelvic plexus through sacrospinous ligament and cardinal ligament. The pains stimulation from pelvic cavity is transmitted to cerebral center to cause pain, suggesting that cutting off relevant nerves can mitigate endometriosis-caused pain.^14^ Thus, during the treatment of deeply infiltrative endometriosis-induced pain, cutting off invaded sacrospinous ligament or blocking relevant nerves can effectively alleviate clinical pain. However, the hypogastric nerve plexus around sacrospinous ligament should not be injured, aiming to prevent complications such as rectal and urinary bladder dysfunction.

In this study, the postoperative pain of observation group (pain VAS score) was relieved significantly, that the VAS scores in the two groups were significantly lower than those before the operation, and the VAS score in the observation group was significantly lower than that of the control group. It indicated that the sacral ligamenttomy on the basis of the conventional operation could significantly improve the pain, which was consistent with the results of the Fermaut M^15^ et al. And urinary bladder or rectal functions were not influenced due to reservation of the hypogastric nerve. However, the two groups had significantly different operation durations and bleeding amounts during operation, indicating that adhesion separation and sacrospinous ligament excision in the operation of observation group may extend the operation time, with larger bleeding amount than that of the control group. Thus, sufficient blood should be prepared before operation. Moreover, it is necessary to further improve the skills of laparoscopic surgery.

In conclusion, deeply infiltrative endometriosis-caused pain can be effectively treated with sacrospinous ligament excision under laparoscope. Regardless, the long-term effects need to be further verified through increasing the number of clinical cases and extending the observation time.

### Authors’ Contributions

**JL, XY & JZ:** Designed this study and prepared this manuscript.

**XY & JZ:** Performed this study.

**JL & XY:** Collected and analyzed clinical data.
